# A Pilot Detection and Associate Study of Gene Presence-Absence Variation in Holstein Cattle

**DOI:** 10.3390/ani14131921

**Published:** 2024-06-28

**Authors:** Clarissa Boschiero, Mahesh Neupane, Liu Yang, Steven G. Schroeder, Wenbin Tuo, Li Ma, Ransom L. Baldwin, Curtis P. Van Tassell, George E. Liu

**Affiliations:** 1 Animal Genomics and Improvement Laboratory, BARC, Agricultural Research Service, USDA, Beltsville, MD 20705, USAsteven.schroeder@usda.gov (S.G.S.); ransom.baldwin@usda.gov (R.L.B.VI);; 2 Department of Veterinary Medicine, University of Maryland, College Park, MD 20742, USA; 3 Department of Animal and Avian Sciences, University of Maryland, College Park, MD 20742, USA; lima@umd.edu; 4 Animal Parasitic Diseases Laboratory, BARC, Agricultural Research Service, USDA, Beltsville, MD 20705, USA; wenbin.tuo@usda.gov

**Keywords:** Holstein, genome, presence-absence variation (PAV), olfactory receptor

## Abstract

**Simple Summary:**

PAV, or presence-absence variation, means that some individuals have certain genes, while others of the same species do not. This study analyzed PAVs in 173 Holstein bulls using whole-genome sequencing and examined their associations with 46 traits. Out of 28,772 genes, an average of 98.06% were present and 1.94% were absent. A total of 1793 genes were missing in at least one bull, including olfactory receptor (OR) genes, other protein-coding genes, tRNAs, microRNAs, and uncharacterized genes. Core genes (present in all bulls) made up 93.77% of the gene pool, while variable genes included softcore (present in 95–99%), shell (5–94%), and cloud genes (<5%). Cloud genes were linked to hormonal and antimicrobial functions, and shell genes to immune functions. Genetic analysis showed high similarity among the bulls, with few outliers. PAV-based genome-wide association studies (GWAS) found links between PAVs and 15 traits, including milk, fat, and protein yields, health (metritis), and reproduction traits, particularly on chromosomes 15 and 7, involving OR and immune-related genes. This research provides insights into the genetic structures underlying complex traits in Holstein cattle. These findings fill gaps in our understanding and lay the groundwork for integrating PAV into future animal breeding programs as a prediction tool.

**Abstract:**

Presence-absence variations (PAVs) are important structural variations, wherein a genomic segment containing one or more genes is present in some individuals but absent in others. While PAVs have been extensively studied in plants, research in cattle remains limited. This study identified PAVs in 173 Holstein bulls using whole-genome sequencing data and assessed their associations with 46 economically important traits. Out of 28,772 cattle genes (from the longest transcripts), a total of 26,979 (93.77%) core genes were identified (present in all individuals), while variable genes included 928 softcore (present in 95–99% of individuals), 494 shell (present in 5–94%), and 371 cloud genes (present in <5%). Cloud genes were enriched in functions associated with hormonal and antimicrobial activities, while shell genes were enriched in immune functions. PAV-based genome-wide association studies identified associations between gene PAVs and 16 traits including milk, fat, and protein yields, as well as traits related to health and reproduction. Associations were found on multiple chromosomes, illustrating important associations on cattle chromosomes 7 and 15, involving olfactory receptor and immune-related genes, respectively. By examining the PAVs at the population level, the results of this research provided crucial insights into the genetic structures underlying the complex traits of Holstein cattle.

## 1. Introduction

Holstein cows hold significant economic importance in the dairy industry worldwide due to their high efficiency in converting feed into milk. In recent decades, a substantial amount of cattle short-read whole-genome sequence (WGS) data have been generated from different breeds in various geographical locations and environmental conditions [1,2]. For example, cattle WGS data have frequently been employed to identify single-nucleotide polymorphisms (SNPs) and insertions/deletions (INDELs) for population genetics and genome-wide association studies [3,4,5,6,7]. They have also been utilized to investigate CNVs [8,9,10,11,12] and SVs [3,13,14,15,16]. The advent of long-read sequencing technologies has enabled the detection of SVs. However, their utility at the population scale is hindered by their high cost, limited throughput, and requirements for large quantities of DNA [17].

Structural variations (SVs) are genomic alterations larger than 50 bp and include copy number variations (CNVs) and rearrangements (inversions and translocations) [18,19]. Presence-absence variations (PAVs) represent a specific form of SV, wherein a genomic segment contains one or more genes that are present in some individuals but absent in others [20]. As many PAVs are identified in different species, their occurrence differs across populations and individuals. PAVs are crucial for phenotypic diversity, population adaptation, and evolution studies [21]. Therefore, PAVs can help to elucidate certain portions of heritability not captured by SNP-based genome-wide association studies (GWAS) [22].

According to the pangenome definition, the core genome comprises genes present in all individuals, whereas the dispensable genome encompasses unique genes specific to some individuals [21,23]. The core and dispensable genomes can vary between closely related species and even among individuals of the same species. The dispensable genome consists of genes with small or rare effects [24], yet they may have significant evolutionary functions and contribute to phenotypic variation [21,25]. For instance, in the mussel genome, approximately 38% of the genome was identified as dispensable [26]. In chickens, the dispensable genome was ~24% when analyzing 664 individuals from different breeds, showing a moderate core gene content of ~76% [27]. In pigs, the dispensable genome was ~17% when analyzing three European commercial breeds and 18 Chinese domestic breeds [28].

Despite extensive research on PAVs in plants [29,30,31,32,33], as well as in other species such as mussels [26,34], chickens [27], and pigs [28], reports on PAVs in cattle are scarce. Moreover, while these data were primarily utilized for SNP and CNV detection, examination of gene PAVs among Holstein dairy cattle has not been reported. The objective of this study was to utilize WGS data from 173 Holstein dairy cattle to identify gene PAVs at the population level. Subsequently, association analyses were conducted to assess their correlation with economically significant traits and to gain insights into their underlying genetic structure.

## 2. Methods

### 2.1. WGS Sequence, Preprocessing, and Alignment

From a previous publication [35], we retrieved 173 items of WGS data from registered Holstein bulls generated using an Illumina short-read sequencing platform. In that study, the authors selected animals by representing most of the haplotypes in the entire registered US dairy herd [35]. All raw reads underwent quality assessment using FastQC (version 0.11.9) (http://www.bioinformatics.babraham.ac.uk/projects/fastqc/ accessed on 17 January 2023). Clean reads were obtained by removing adaptors and low-quality reads with Trimmomatic (version 0.38) [36] using the following parameters: TruSeq3-PE.fa:2:30:10, LEADING:3, TRAILING:3, SLIDINGWINDOW:4:15, and MINLEN:36. Subsequently, reads were mapped to the cattle ARS-UCD1.3 reference genome [37] using BWA-MEM with the default parameters (version 0.7.17) [38]. PCR duplicate reads were removed using Picard (version 3.0.0) [39]. BAM files were generated after mapping and processed using SAMtools (version 1.17) [40]. Finally, the sequence depth was obtained for each sample with Mosdepth (version 3.0.0) [41].

### 2.2. Phenotypes, dPTA, and Correlation Analysis

Individual trait data for 154 registered Holstein bulls were retrieved from the National Cooperator Database from the U.S. Council of Dairy Cattle Breeding (CDCB). These data were part of the December 2022 genomic evaluations from the CDCB, which routinely calculates predicted transmitting ability (PTA) values for dairy cattle of different breeds. These 46 phenotypes are described in Supplementary Table S1.

De-regressed PTA (dPTA) from all traits were used for PAV-based GWAS and calculated according to the formula: dPTA = PTA/reliability, as previously described [42]. Pearson correlations were computed between pairs of dPTA for all 46 phenotypes using R for 154 Holstein bulls.

### 2.3. Gene Presence-Absence Variation Identification

SGSGeneLoss (version 0.1) was used to identify the presence and absence of genes in each sample [43,44]. The parameters used were minCov = 5 and lostCutoff = 0.2. A PAV was classified as present if it had more than 20% coverage with at least five reads; otherwise, it was classified as absent. The cattle transcripts (66,384) were filtered to keep only the longest transcript for each of the genes, resulting in 28,772 genes. Following PAV identification, the present genes were classified as follows: core (present in 100% of the accessions), softcore (present in 95–99%), shell (present in 5–94%), and cloud (present in <5%) genes, based on their gene presence frequencies [27,33].

### 2.4. Statistical Overrepresentation Tests of Variable Genes

We conducted a statistical overrepresentation analysis using PANTHER (version 18.0) [45] with the PANTHER Gene Ontology (GO) slim datasets for Biological Process (BP), Molecular Function (MF), Cellular Component (CC), and Reactome. Fisher’s exact test, adjusted for the false discovery rate (FDR < 0.05), was employed for this analysis. Specifically, the test was conducted for each variable gene category (softcore, shell, and cloud) to ascertain if there was a significant overrepresentation of any gene category.

### 2.5. Population Genetic Analyses

The identified gene presence-absent variations were further employed in population genetic analyses. We constructed a PAV matrix for the 173 animals and 28,772 genes, followed by principal component analysis (PCA) and neighbor-joining phylogenetic analysis conducted using TASSEL (version 5.0) [46].

### 2.6. Gene PAV-Based GWAS

PAV-based GWAS was conducted with 46 traits (dPTA), including production, body type, reproduction, and health traits from 154 animals (Supplementary Table S1). Prior to analysis, PAVs were filtered based on a Minor Allele Frequency (MAF) > 0.05 and chromosome range (bovine autosomes). The association analysis was performed using TASSEL (version 5.0) [46], employing the general linear model (GLM). A Bonferroni test was applied to establish the genome-wide significance (0.05/number of loci) or suggestive (0.1/number of loci) cut-off thresholds.

## 3. Results

### 3.1. Phenotype Correlation

**Figure 1:**
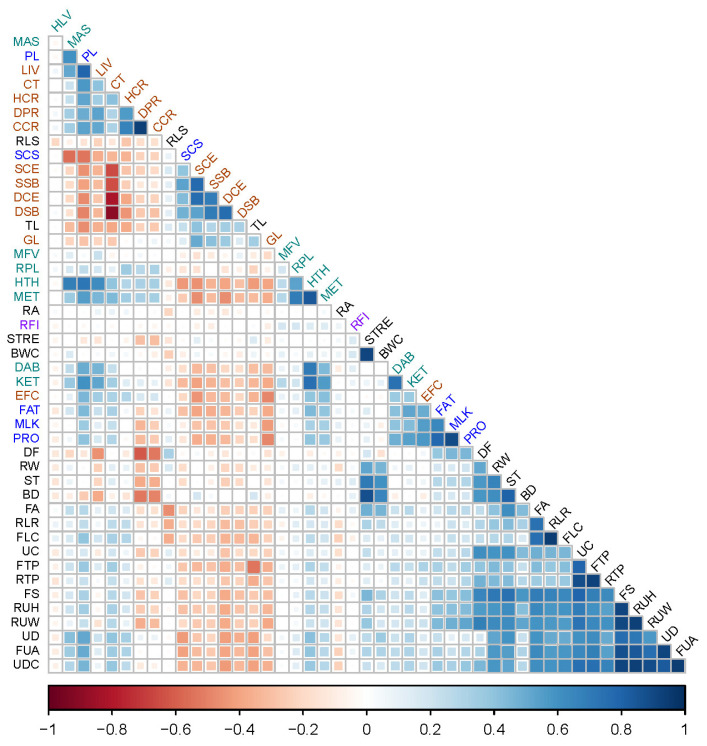
Pairwise Pearson correlation coefficients between each of the 46 dairy phenotypes based on the de-regressed PTA (dPTA). BD: body depth; BWC: body weight composite; CT: calving trait composite; CCR: cow conception rate; LIV: cow livability; DF: dairy form; DCE: daughter calving ease; DPR: daughter pregnancy rate; DSB: daughter stillbirth; DAB: displaced abomasum; EFC: early first calving; FAT: fat yield; FLC: feet and leg composite; FS: final score; FA: foot angle; FUA: fore udder attachment; FTP: front teat placement; GL: gestation length; HTH: health trait composite; HCR: heifer conception rate; HLV: heifer livability; KET: ketosis; MAS: mastitis; MET: metritis; MFV: milk fever/hypocalcemia; MLK: milk yield; PL: productive life; PRO: protein yield; RLR: rear legs (rear view); RLS: rear legs (side view); RTP: rear teat placement; RUH: rear udder height; RUW: rear udder width; RFI: residual feed intake; RPL: retained placenta; RA: rump angle; RW: rump width; SCE: sire calving ease; SSB: sire stillbirth; SCS: somatic cell score; ST: stature; STRE: strength; TL: teat length; UC: udder cleft; UDC: udder composite; UD: udder depth. The color bar on the bottom represents the r values ranging from −1 (red) to 1 (blue). Traits’ categories have different colors: Body Type (black); Health (green); Feed Efficiency (purple); Production (blue); and Reproduction (dark red).

Figure 1 shows the results of the Pearson correlation analysis between each pair of the dPTA. We observed moderate to strong correlations within phenotypic groups, for example, for body type traits, FUA vs. UD (r = 0.915, *p* < 0.0001), FTP vs. RTP (r = 0.924, *p* < 0.0001), UDC vs. FUA (r = 0.942, *p* < 0.0001), and RUH vs. FS (r = 0.917, *p* < 0.0001) (Supplementary Table S2). We also detected moderate and high correlations among production (FAT, MLK, and PRO), reproduction (PL, LIV, CT, HCR, DPR, and CCR), and health traits (MAS, MET, KET, DAB, RPL, and HTH). For example, MLK versus PRO and MLK versus FAT were highly correlated (r > 0.6). On the other hand, calving traits such as SCE, DCE, SSB, DSB, and SCS are negatively correlated with most traits in the other groups. In addition, productive life is moderately correlated with fertility phenotypes (DPR, CT, HCR, CCR, and EFC). Somatic cell score showed a moderate to weak negative correlation with other traits, such as PL (r = −0.539, *p* < 0.0001), HCR (r = −0.340, *p* < 0.0001), and CT (r = −0.349, *p* < 0.0001).

### 3.2. Mapping Reads

**Figure 2:**
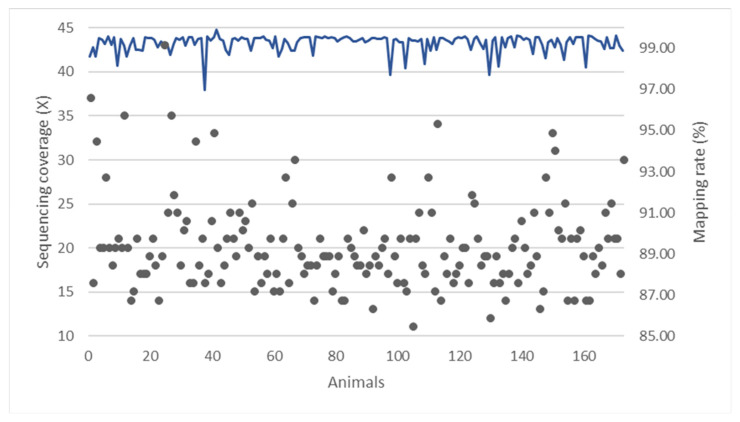
The sequencing coverage (×) and mapping rate (%) across 173 Holstein animals. Black dots represent the sequencing coverage, and blue line represents the mapping rate.

The sequencing data for samples of 173 Holstein cattle comprised paired-end reads with a maximum length of 151 bp, yielding an average of 617,227,322 raw reads for each sample (Supplementary Table S3). Following the filtering process, an average of 556,623,661 clean reads were obtained, representing an average of 90.18% of the raw data that were used for subsequent analysis (Supplementary Table S3). The clean reads were then aligned to the *B. taurus* ARS-UCD1.3 reference genome assembly [37], resulting in an overall average mapping rate of 99.26% across all 173 samples (Figure 2) This alignment produced an average of 0.20% of singleton reads, 99.07% of paired mapped reads, and 0.74% of unmapped reads (Supplementary Table S3).

The average sequencing coverage for all 173 samples was 20.13×, and no samples showed less than 10× coverage (Supplementary Table S3, Figure 2). Out of these, 65 samples exhibited coverage greater than 20× (38%), and 150 samples showed a coverage exceeding 15× (87%).

### 3.3. Identification of Gene PAVs in Holstein Cattle

**Figure 3:**
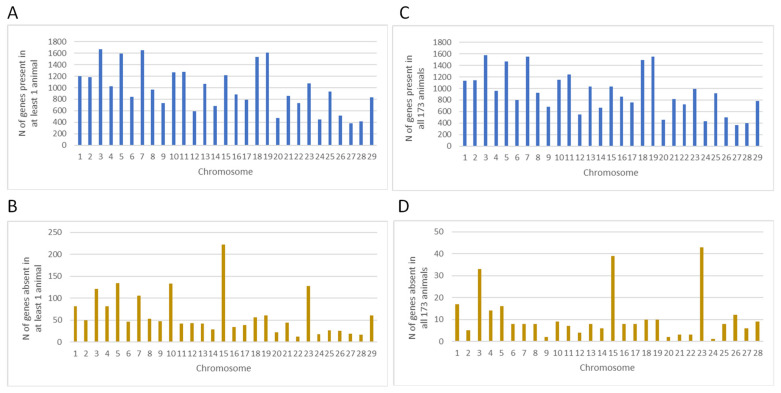
The distribution of gene PAVs across bovine autosomes in 173 Holstein cattle. (**A**) Gene PAVs present in at least one animal. (**B**) Gene PAVs absent in at least one animal. (**C**) Gene PAVs present in all 173 animals. (**D**) Gene PAVs absent in all 173 animals.

Gene PAVs were identified for each sample across bovine autosomes. A total of 28,772 cattle genes (with only the longest transcripts included) were assessed by PAV analysis. A gene was considered present if at least five reads have covered more than 20% of the cumulative coverage of the exons of each gene, as defined previously [27,33]. Otherwise, the gene was considered absent. Based on this definition and considering the 173 animals, on average, 28,214 genes (98.06%) were present in a particular animal, while 558 genes were absent (1.94%) (Supplementary Table S4). Among the 28,772 cattle genes, 28,465 genes were present in at least one animal, and 26,979 genes were present in all 173 Holstein animals (Supplementary Table S5). The chromosome distribution of the genes present exhibited a similar pattern when the genes were present in at least one animal vs. present in all 173 animals (Figure 3). BTA3, 7, 19, 5, and 18 harbored a higher number of genes present compared to all other chromosomes.

The absent genes in at least one individual animal were merged to create a comprehensive list of all dispensable genes (Supplementary Table S6). A total of 1793 genes were absent in at least one animal, comprising ~69% of uncharacterized genes, ~13% of transfer RNA (tRNAs), ~4% of microRNAs, and ~3% of olfactory receptor genes (Supplementary Table S6). Among these, 51 animals (~30%) exhibited ≥ 2.0% absent genes. Additionally, 307 genes were absent in all 173 animals, including 194 uncharacterized genes, six immune-related genes (*IFITM1*, *IFNB1*, *IFNL3*, *IFNT3*, *CATHL1*, and *CATHL4*), eight olfactory receptor genes (*OR10AD1*, *OR2AG1*, *OR52J3*, *OR6C3*, etc.), 70 tRNAs genes, one reproduction-related gene (*GPX5*), and other genes including *BMP8A*, *GML*, *HBA*, *HBA1*, *KYAT1*, *TAP2*, *TUBA3E,* and *WDR36*. The chromosome distribution of absent genes exhibited a similar pattern whether the gene was absent in at least one animal or all 173 animals (Figure 3). A higher number of genes were absent on BTA15, 5, 10, 23, and 3. BTA15 displayed the highest number of genes absent in at least one animal compared to the other chromosomes, with approximately 80% being uncharacterized genes, and 12.6% being olfactory receptor genes (Figure 3B).

**Figure 4:**
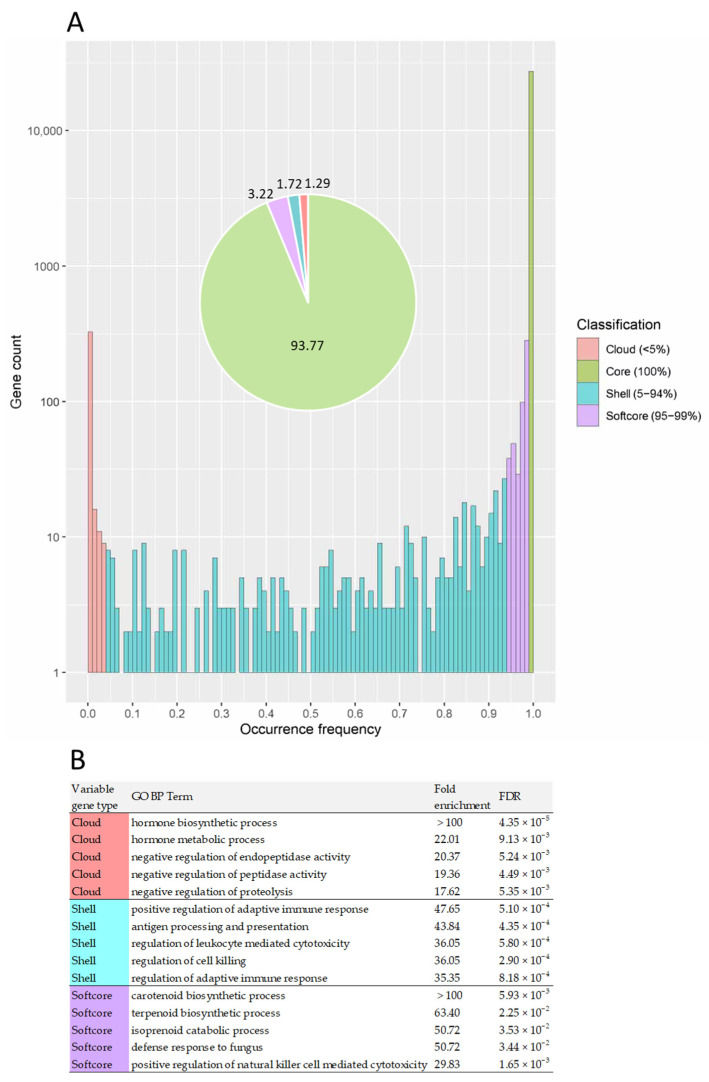
(**A**) Gene classification in 173 Holstein cattle according to their gene presence frequencies. The present genes were classified as core (present in 100% of the individuals), softcore (present in 95–99%), shell (present in 5–94%), and cloud (present in <5%) genes. (**B**) The top five Biological Process (BP) GO terms are overrepresented in the variable genes.

Additionally, the present genes were classified as core, softcore, shell, and cloud genes present in 100%, 95–99%, 5–94%, and <5% of all animals, respectively. A total of 26,979 (93.77%) core genes are shared by all 173 individuals. Furthermore, 1793 genes are variable (i.e., missing in at least one individual), including 928 softcore, 494 shell, and 371 cloud genes (Figure 4A). Statistical overrepresentation analysis (including GO and Reactome) results revealed the enrichment of variable genes, as shown in Supplementary Table S7. Cloud genes were significantly enriched (FDR < 0.05) in functions associated with hormone and antimicrobial activities, glucocorticoid biosynthesis, regulation of peptidase activity, lipopolysaccharide binding, and antioxidant activity (Figure 4B, Supplementary Table S7). Shell genes were predominantly enriched (FDR < 0.05) with immune system functions (Figure 4B, Supplementary Table S7). Softcore genes showed enrichment (FDR < 0.05) in functions associated with carotenoid and terpenoid biosynthesis, the immune system, regulation of the receptor signaling pathway via JAK-STAT, DAP12, cell communication and signaling, sensory perception, and response to chemicals or stimuli (Figure 4B, Supplementary Table S7).

### 3.4. PAV Analysis

PCA and neighbor-joining phylogenetic analysis were performed to assess gene diversity based on the identified gene PAVs. Gene PAV-based PCA and phylogenetic analysis revealed high similarity among the 173 Holstein animals (Supplementary Figure S1). Specifically, PCA indicated that seven animals clustered separately from the rest (Supplementary Figure S1A), while the neighbor-joining phylogenetic tree also showed high similarity, with 10 animals exhibiting slightly more divergence (Supplementary Figure S1B).

### 3.5. Gene PAV-Based GWAS

PAV-based GWAS was conducted with 46 traits from 154 animals using TASSEL [46] and GLM analysis to explore the traits associated with gene PAVs (Supplementary Table S1). Filtering based on a minor allele frequency (MAF) threshold of >0.05 resulted in 487 markers for analysis. We identified 18 associations (six significant and 12 suggestive) involved in 16 traits including CCR, CT, DCE, EFC, FAT, FLC, FS, FUA, HTH, KET, LIV, MET, MFV, MLK, PL, and PRO (Table 1).

PAV-based GWAS identified eight associations on BTA15 with three olfactory receptor genes associated with seven traits. On BTA14, *LOC112449566* (*cytochrome P450 11B1*, mitochondrial-like) was associated with PL and FUA. On BTA7, one association was identified with PL (*LOC100337044* or adhesion G protein-coupled receptor E3). On BTA19, *LOC112442670* (keratin-associated protein 9-7-like) was associated with MFV. Notably, two immune-related genes were associated with LIV (*LOC100296997* or T cell receptor alpha variable 14/delta variable 4-like) and FLC (*LOC789175* or beta-defensin 103B-like).

## 4. Discussion

In this study, we conducted an analysis of sequencing data obtained from 173 Holstein bulls, aiming to identify the PAVs in dairy cattle for the first time. Our analysis revealed gene presence variations across the sampled population. Notably, all animals exhibited a high average mapping rate, with a moderate sequencing coverage of 20×, where 87% of the bulls had a coverage exceeding 15×. A previous study on PAV in chickens utilized a minimal sequencing coverage cut-off of less than 10× [27]. Prior research has demonstrated that a sequencing coverage of 10× allows for the recovery of between 98–99.9% of gene PAVs [27,33].

Among the 28,772 cattle genes, the majority were classified as present (>20% of coverage with at least five reads in one animal), with an average of 28,214 present genes (98.06%), while an average of 558 genes were identified as absent. Impressively, 93.77% of these genes, totaling 26,979, were present in all 173 Holstein animals, indicating a substantial proportion of core genes compared to other studies [9,10,34]. For example, a study in chickens identified 76% of core genes [27], but unlike our study that analyzed only one cattle breed (Holstein), this chicken study evaluated 664 individuals from five wild subspecies with 28 native breeds and four commercial breeds. Similarly, a study in mussels identified 69% of core gene content from different European populations [26]. A study in pigs identified 83.2% of core genes shared by all individuals from three European commercial breeds and 18 Chinese domestic breeds [28]. The high levels of core genes observed in our study may be attributed to our focus on a single cattle breed (Holstein), which likely shares a similar genetic background and exhibits high genetic homology.

Despite the predominance of core genes, we identified 1793 variable or dispensable genes (6.24%) across the 173 bulls. While this proportion seems relatively small, of the 28,772 genes, these genes could signify crucial genomic regions contributing to cattle genetic variability and potentially be indicative of environmental adaptation. Comparable findings in humans suggest that about 10% of genes are dispensable [47,48], yet they may play significant roles in genome evolution or phenotypic variation, as demonstrated in previous studies [21,25,49].

The differentiation between core and dispensable genome components is not static, and the genome may undergo alterations due to PAVs or SVs, leading to changes in the proportions of the genome classes and potentially creating a conditionally dispensable genome [21]. Although our study revealed a lower content of dispensable genes compared to humans [47,48], chickens [27], mussels [26], or pigs [28], a previous study in cattle reported a similar dispensable genome content when analyzing the whole genome of five cattle assemblies from Angus, Highland, and Original Braunvieh, and their close relative Brahman [50]. That study observed that 6.10% of the cattle genome is dispensable (or flexible), albeit without identifying PAVs [50]. These findings collectively underscore the importance of understanding both core and dispensable genome components in elucidating genetic diversity and evolutionary dynamics in cattle populations.

In our analysis of 173 Holstein cattle, we identified 1793 variable genes that were not universally present in all individuals. These genes were categorized based on their frequency of occurrence: cloud genes (<5%), shell genes (5–94%), and softcore genes (95–99%). Cloud genes, characterized by their rare occurrence (<5%), were notably enriched in functions related to hormone activity, peptidase activity, regulation of proteolysis, and glucocorticoid biosynthesis. Shell genes, which exhibited intermediate frequency (5–94%), were predominantly associated with immune-related functions. Softcore genes, occurring with relatively higher frequency (95–99%), showed enrichment in various functions including immune-related functions, regulation of the JAK-STAT signaling cascade, DAP12 signaling, response to nutrient levels, sensory perception, and others. Importantly, softcore genes showed enrichment in functions related to the innate immune response. Of particular interest is the DAP12 signaling pathway, which is known for its role in innate immunity responses [51]. In our study, genes enriched for this pathway included *KLRC1*, *NKG2A*, and *NRAS.* Additionally, the cytokine-activated Janus kinase (JAK)/signal transducer and activator of transcription (STAT) pathway, crucial in modulating immunity and inflammation, has been linked to mastitis resistance and milk production in dairy cattle [52]. In our findings, genes associated with this pathway were *PRP1* (prolactin-related protein 1), *PRP2*, and *PRP14*, suggesting potential implications for immune response and milk production traits.

In addition, softcore genes were identified within functions related to sensory perception (GO:0007600), responses to chemicals (GO:0042221), and the detection of stimulus (GO:0051606). GO BP term pathways were mainly olfactory receptors (ORs) and taste receptor genes. ORs constitute the largest gene family within the mammalian genome, with ~400 functional genes (with intact coding regions) and over 400 pseudogenes (nonfunctional segments) in humans [53,54,55]. The composition of OR members vary significantly across species and individuals due to the abundance of pseudogenes, CNVs, deletions, and SNPs [55,56,57]. In cattle, 1071 OR genes have been identified, including 881 functional genes, 190 pseudogenes, and 352 partial genes distributed across 26 chromosomes, with BTA15 harboring the highest number of functional genes (251) [58]. In our study, a total of 225 OR genes were identified, of which 165 OR are core genes and 62 are variable genes, including 35 softcore, eight cloud, and 19 shell genes. Our analysis revealed that 10 OR genes were absent in all 173 Holstein animals studied. Given that one odor can activate multiple ORs, there certainly exists a degree of redundancy among OR genes. Studies have demonstrated that even subtle functional changes in specific OR genes can profoundly alter odor perception in humans [59,60,61], underscoring the significant impact a single OR may have on odorant perception.

In this study, cattle PAV-based GWAS was performed with 46 phenotypes. Phenotype correlation analysis indicated moderate to strong correlations within each trait group, as described before in cattle [62,63]. The gene PAV-based GWAS analysis yielded 18 associations between gene PAVs and traits in dairy cattle. Notably, several OR genes, including *OR52N2*, *OR52E6*, *LOC785207*, and *LOC782221*, showed associations with several important correlated traits. *OR52E6* was linked to MET, HTH, FAT, and EFC, while *OR52N2* was associated with PRO, MLK, and MET. *LOC785207* (olfactory receptor family 52 subfamily S member 2) was found to be associated with FS, while *LOC782221* (olfactory receptor family 8 subfamily B member 1AQ), despite being a pseudogene (being recently updated), showed an association with CT. Although pseudogenes are generally considered non-functional, a human GWAS study revealed associations of 13 pseudogenes with disease susceptibility [64]. Despite encoding truncated proteins, pseudogenes can still be transcribed into RNA and may play a role in regulating gene expression [65]. Although significant sites may only indicate markers linked to the causative mutation, there is a notable enrichment of pseudogenes and nonfunctional genes within the OR gene repertoire [57].

These associated OR genes belong to the OR52 family, while one is from the OR8 family. Subtypes of the OR52 family in humans are known to recognize carboxylic acids [66], as well as butter-like aromas (butanoic acid, gamma decalactone, and diacetyl) [67]. Odor perception is crucial for cattle, influencing their selection of feed, avoidance of dangers, and engagement in reproductive and social behaviors. The high variability observed in the OR gene repertoire in cattle may be attributed to selection pressures and environmental changes [58]. In humans, OR genes have been linked to appetite regulation and food intake [68,69]. Similarly, in cattle, OR genes have been associated with diverse economic traits such as feed intake [63,70], feed utilization [71], reproduction [72], carcass performance [73], and methane emission [74]. This suggests a potential regulation of appetite in cattle by ORs, which could impact feed intake, feeding behavior, body composition, and weight gain [70].

Moreover, our gene PAV-GWAS results showed that *LOC112442670* (keratin-associated protein 9-7-like) was associated with MFV in dairy cattle. Furthermore, two other immune genes were associated with important traits in dairy cattle. *LOC100296997* (T cell receptor alpha variable 14/delta variable 4-like) is associated with LIV, and *LOC789175* (beta-defensin 103B-like) is linked to the FLC. β-defensins, prominent antimicrobial peptides, serve as the primary defense against prevalent infections in dairy cattle, including intramammary infections [75]. Additionally, β-defensins contribute to regulating epithelial proliferation during the wound-healing process [76].

The *LOC112449566* gene (cytochrome P450 11B1, mitochondrial-like) was found to be associated with PL and FUA in dairy cattle. A previous study in dairy cows has identified associations of *LOC112449566* with lactose yield in Fleckvieh cattle [77]. Additionally, in a recent study of milk production traits, this gene was also associated with fat percentage in Walloon Holstein cows [78]. These findings suggest that *LOC112449566* is a potential candidate gene in cattle for further investigation into its role in milk production and related traits.

In our study, we also found one adhesion G protein-coupled receptor gene, *LOC100337044*, located on BTA7, was associated with PL in dairy cattle. G protein-coupled receptors (GPCRs) are cell surface receptors that detect molecules outside the cell, comprising over 750 members in mammals [79]. Adhesion G protein-coupled receptors (AGPCRs) constitute a subgroup of GPCRs, consisting of 33 members in humans with diverse expression patterns and functions, which are primarily involved in the regulation of adhesion [80]. In humans, the *ADGRE3* (adhesion G protein-coupled receptor E3) or *EMR3* gene is predominantly expressed by cells of the immune system and plays a role in leukocyte migration [81]. A study evaluating differences in the innate immune response in Holstein and Angus cattle found varying levels of methylation in the *LOC100337044* gene in fibroblast cultures challenged with *E. coli* lipopolysaccharide [82]. Additionally, another study in cattle identified the *LOC100337044* gene associated with protein percentage in Thai dairy cattle [83]. These findings underscore the potential significance of AGPCR genes in dairy cattle and warrant further investigation into their roles in various physiological processes and traits.

It is imperative to acknowledge that our ability to detect PAVs was constrained by the limitations of both short-read sequencing and the current linear reference genome assembly. However, advancements such as telomere-to-telomere assembly, bovine pangenome graph construction, and the utilization of long-read sequencing technologies hold promise for uncovering PAVs in previously inaccessible genomic regions. These advancements will undoubtedly enhance our ability to capture a comprehensive spectrum of PAVs in the future. Moreover, conducting functional validation experiments on the identified candidate genes will be essential for future studies to corroborate our findings.

## 5. Conclusions

The discovery of PAVs in the Holstein bulls has provided novel insights into the genomic landscape of cattle. Our analysis revealed that nearly 98% of assessed genes were present in the population, while approximately 2% exhibited absence variation, indicating underlying genetic diversity. Core genes, found in all individuals, comprised a substantial portion of all genes; the presence of variable genes such as softcore, shell, and cloud genes suggests genomic adaptability. The enrichment of cloud genes in functions such as hormone activity and antimicrobial peptides implies their role in physiological adaptation, while shell genes enriching immune functions highlight the importance of genetic variability in immune traits and host defense. Despite some genomic variability, the overall genetic similarity suggests homogeneity within the population. Furthermore, in this study, we performed a PAV-based GWAS for 46 important production traits, revealing six PAVs located on BTA7, 14, and 15, which were significantly associated with five traits. These significant associations illustrate the potential impact of candidate genes related to OR, AGPCR, and cytochrome genes on vital production and health traits in Holstein dairy cattle. These findings offer valuable insights for future breeding and management strategies aimed at improving breed performance and health outcomes.

## Figures and Tables

**Table 1:** Summary of the most significant gene PAV effects on 46 dairy traits in Holstein cattle.

Chr	Position	Gene	Gene Name	Trait and Effect Ranking	*p*-Value	F-Value
Significant Effect						
7	17,361,304	LOC100337044	Adhesion G protein-coupled receptor E3	#1 PL	5.99 × 10^−5^	17.05
14	1,600,535	LOC112449566	Cytochrome P450 11B1, mitochondrial-like	#2 PL	1.01 × 10^−4^	15.96
15	47,227,535	OR52E6	Olfactory receptor family 52 subfamily E member 6	#1 MET	2.10 × 10^−6^	24.34
15	47,227,535	OR52E6	Olfactory receptor family 52 subfamily E member 6	#1 FAT	8.66 × 10^−6^	21.21
15	47,245,425	OR52N2	Olfactory receptor family 52 subfamily N member 2	#1 MLK	4.41 × 10^−6^	22.69
15	47,245,425	OR52N2	Olfactory receptor family 52 subfamily N member 2	#1 PRO	1.38 × 10^−5^	20.20
Marginal Effect						
3	54,809,083	LOC785445	Heterogeneous nuclear ribonucleoprotein A1-like	#1 DCE	1.90 × 10^−4^	14.63
10	22,785,310	LOC100296997	T cell receptor alpha variable 14/delta variable 4-like	#1 LIV	1.61 × 10^−4^	14.97
14	1,600,535	LOC112449566	Cytochrome P450 11B1, mitochondrial-like	#1 FUA	1.60 × 10^−4^	14.99
15	47,227,535	OR52E6	Olfactory receptor family 52 subfamily E member 6	#1 HTH	1.05 × 10^−4^	15.86
15	47,227,535	OR52E6	Olfactory receptor family 52 subfamily E member 6	#1 EFC	1.67 × 10^−4^	14.91
15	47,245,425	OR52N2	Olfactory receptor family 52 subfamily N member 2	#2 MET	1.12 × 10^−4^	15.73
15	49,043,281	LOC785207	Olfactory receptor family 52 subfamily S member 2	#1 FS	1.33 × 10^−4^	15.38
18	57,253,674	LOC112442406	Zinc finger protein 85-like (withdrawn by NCBI)	#1 KET	1.61 × 10^−4^	14.98
19	41,468,220	LOC112442670	Keratin-associated protein 9-7-like	#1 MFV	1.12 × 10^−4^	15.74
20	6,875,546	LOC104975198	Uncharacterized (withdrawn by NCBI)	#1 CCR	1.85 × 10^−4^	14.69
27	6,023,993	LOC789175	beta-defensin 103B-like	#1 FLC	1.52 × 10^−4^	15.10
29	27,350,311	LOC782221	Olfactory receptor family 8 subfamily B member 1AQ	#1 CT	2.01 × 10^−4^	14.52

CCR: cow conception rate; CT: calving trait composite; DCE: daughter calving ease; EFC: early first calving; FAT: fat yield; FLC: feet and leg composite; FS: final score; FUA: fore udder attachment; HTH: health trait composite; KET: ketosis; LIV: cow livability; MET: metritis; MFV: milk fever/hypocalcemia; MLK: milk yield; PL: productive life; PRO: protein yield.

## Data Availability

The original phenotype data are owned by third parties and maintained by the Council on Dairy Cattle Breeding (CDCB). A request to CDCB is necessary for getting data access on research, which may be sent to: João Dürr, CDCB Chief Executive Officer (joao.durr@cdcb.us). All other data have been included in the manuscript and Supplementary Materials.

## Supplementary Materials

The following supporting information can be downloaded at: https://www.mdpi.com/article/10.3390/ani14131921/s1. Supplementary Figure S1. (A) Principal component analysis based on gene PAV matrix of 173 Holstein animals. (B) Neighbor-joining phylogenetic tree constructed based on gene PAV matrix of 173 Holstein animals. Supplementary Table S1. (A) Description of the 46 phenotypes from five groups (Body Type; Health; Feed Efficiency; Production; and Reproduction) in Holstein cattle used for gene PAV-GWAS showing their respective reliabilities. (B) Trait list from each group. Body Type (B); Health (H); Feed Efficiency (E); Production (P); and Reproduction (R). Supplementary Table S2. (A) Pairwise Pearson correlation coefficients for 46 dairy phenotypes based on the de-regressed PTA (dPTA) and (B) respective p-values. BD: body depth; BWC: body weight composite; CT: calving trait composite; CCR: cow conception rate; LIV: cow livability; DF: dairy form; DCE: daughter calving ease; DPR: daughter pregnancy rate; DSB: daughter stillbirth; DAB: displaced abomasum; EFC: early first calving; FAT: fat yield; FLC: feet and leg composite; FS: final score; FA: foot angle; FUA: fore udder attachment; FTP: front teat placement; GL: gestation length; HTH: health trait composite; HCR: heifer conception rate; HLV: heifer livability; KET: ketosis; MAS: mastitis; MET: metritis; MFV: milk fever/hypocalcemia; MLK: milk yield; PL: productive life; PRO: protein yield; RLR: rear legs (rear view); RLS: rear legs (side view); RTP: rear teat placement; RUH: rear udder height; RUW: rear udder width; RFI: residual feed intake; RPL: retained placenta; RA: rump angle; RW: rump width; SCE: sire calving ease; SSB: sire stillbirth; SCS: somatic cell score; ST: stature; STRE: strength; TL: teat length; UC: udder cleft; UDC: udder composite; UD: udder depth. Traits’ categories are Body Type (B); Health (H); Feed Efficiency (E); Production (P); and Reproduction (R). Supplementary Table S3. Mapping summary statistics of 173 bull Holstein cattle. Supplementary Table S4. Summary of PAVs identified in the 173 bull Holstein cattle. Supplementary Table S5. List of PAVs identified in the 173 bull Holstein cattle with their respective genes, genomic coordinates, and gene frequencies. The presence genes are coded as “1”, and the absent genes are codified as “0”. Supplementary Table S6. List of dispensable genes identified in the 173 bull Holstein cattle. Supplementary Table S7. Statistical overrepresentation of variable genes with the PANTHER GO-slim datasets for Biological Process (BP), Molecular Function (MF), Cellular Component (CC), and Reactome. This analysis was conducted using Fisher’s exact test, adjusted for the false discovery rate (FDR < 0.05).

## Abbreviations

AGPCR	Adhesion G protein-coupled receptors
BD	body depth
BTA	*Bos taurus* autosome
BWC	body weight composite
CCR	cow conception rate
CDCB	Council of Dairy Cattle Breeding
CNV	copy number variations
CT	calving trait composite
DAB	displaced abomasum
DCE	daughter calving ease
DF	dairy form
DPR	daughter pregnancy rate
dPTA	De-regressed PTA
DSB	daughter stillbirth
EFC	early first calving
FA	foot angle
FAT	fat yield
FLC	feet and leg composite
FS	final score
FTP	front teat placement
FUA	fore udder attachment
GL	gestation length
GLM	general linear model
GPCR	G protein-coupled receptors
GWAS	genome-wide association studies
HCR	heifer conception rate
HLV	heifer livability
HTH	health trait composite
INDEL	insertion/deletion
JAK	cytokine-activated Janus kinase
KET	Ketosis
LIV	cow livability
MAS	Mastitis
MET	Metritis
MFV	milk fever/hypocalcemia
MLK	milk yield
OR	olfactory receptors
PAV	Presence-absence variations
PCA	principal component analysis
PL	productive life
PRO	protein yield
PTA	transmitting ability
RA	rump angle
RFI	residual feed intake
RLR	rear legs (rear view)
RLS	rear legs (side view)
RPL	retained placenta
RTP	rear teat placement
RUH	rear udder height
RUW	rear udder width
RW	rump width
SCE	sire calving ease
SCS	somatic cell score
SSB	sire stillbirth
ST	Stature
STAT	signal transducer and activator of transcription
STRE	Strength
SV	structural variants
TL	teat length
UC	udder cleft
UD	udder depth.
UDC	udder composite

## References

[1] Zimin A.V., Delcher A.L., Florea L., Kelley D.R., Schatz M.C., Puiu D., Hanrahan F., Pertea G., Van Tassell C.P., Sonstegard T.S. (2009). A whole-genome assembly of the domestic cow, Bos taurus. Genome Biol..

[2] Weldenegodguad M., Popov R., Pokharel K., Ammosov I., Ming Y., Ivanova Z., Kantanen J. (2019). Whole-Genome Sequencing of Three Native Cattle Breeds Originating From the Northernmost Cattle Farming Regions. Front. Genet..

[3] Zhou Y., Yang L., Han X., Han J., Hu Y., Li F., Xia H., Peng L., Boschiero C., Rosen B.D. (2022). Assembly of a pangenome for global cattle reveals missing sequences and novel structural variations, providing new insights into their diversity and evolutionary history. Genome Res..

[4] Sun T., Pei S., Liu Y., Hanif Q., Xu H., Chen N., Lei C., Yue X. (2023). Whole genome sequencing of simmental cattle for SNP and CNV discovery. BMC Genom..

[5] Jiang L., Kon T., Chen C., Ichikawa R., Zheng Q., Pei L., Takemura I., Nsobi L.H., Tabata H., Pan H. (2021). Whole-genome sequencing of endangered Zhoushan cattle suggests its origin and the association of MC1R with black coat colour. Sci. Rep..

[6] Peripolli E., Reimer C., Ha N.T., Geibel J., Machado M.A., Panetto J.C.D.C., do Egito A.A., Baldi F., Simianer H., da Silva M.V.G.B. (2020). Genome-wide detection of signatures of selection in indicine and Brazilian locally adapted taurine cattle breeds using whole-genome re-sequencing data. BMC Genom..

[7] Paguem A., Abanda B., Achukwi M.D., Baskaran P., Czemmel S., Renz A., Eisenbarth A. (2020). Whole genome characterization of autochthonous Bos taurus brachyceros and introduced Bos indicus indicus cattle breeds in Cameroon regarding their adaptive phenotypic traits and pathogen resistance. BMC Genet..

[8] Liu G.E., Hou Y., Zhu B., Cardone M.F., Jiang L., Cellamare A., Mitra A., Alexander L.J., Coutinho L.L., Dell’Aquila M.E. (2010). Analysis of copy number variations among diverse cattle breeds. Genome Res..

[9] da Silva J.M., Giachetto P.F., da Silva L.O., Cintra L.C., Paiva S.R., Yamagishi M.E., Caetano A.R. (2016). Genome-wide copy number variation (CNV) detection in Nelore cattle reveals highly frequent variants in genome regions harboring QTLs affecting production traits. BMC Genom..

[10] Yang L., Xu L., Zhu B., Niu H., Zhang W., Miao J., Shi X., Zhang M., Chen Y., Zhang L. (2017). Genome-wide analysis reveals differential selection involved with copy number variation in diverse Chinese Cattle. Sci. Rep..

[11] Lee Y.L., Bosse M., Mullaart E., Groenen M.A.M., Veerkamp R.F., Bouwman A.C. (2020). Functional and population genetic features of copy number variations in two dairy cattle populations. BMC Genom..

[12] Zhou J., Liu L., Reynolds E., Huang X., Garrick D., Shi Y. (2022). Discovering Copy Number Variation in Dual-Purpose XinJiang Brown Cattle. Front. Genet..

[13] Boussaha M., Esquerré D., Barbieri J., Djari A., Pinton A., Letaief R., Salin G., Escudié F., Roulet A., Fritz S. (2015). Genome-Wide Study of Structural Variants in Bovine Holstein, Montbéliarde and Normande Dairy Breeds. PLoS ONE.

[14] Chen L., Chamberlain A.J., Reich C.M., Daetwyler H.D., Hayes B.J. (2017). Detection and validation of structural variations in bovine whole-genome sequence data. Genet. Sel. Evol..

[15] Gao Y., Ma L., Liu G.E. (2022). Initial Analysis of Structural Variation Detections in Cattle Using Long-Read Sequencing Methods. Genes.

[16] Leonard A.S., Crysnanto D., Fang Z.H., Heaton M.P., Vander Ley B.L., Herrera C., Bollwein H., Bickhart D.M., Kuhn K.L., Smith T.P.L. (2022). Structural variant-based pangenome construction has low sensitivity to variability of haplotype-resolved bovine assemblies. Nat. Commun..

[17] Ashley E.A. (2016). Towards precision medicine. Nat. Rev. Genet..

[18] Feuk L., Carson A.R., Scherer S.W. (2006). Structural variation in the human genome. Nat. Rev. Genet..

[19] Sharp A.J., Cheng Z., Eichler E.E. (2006). Structural variation of the human genome. Annu. Rev. Genom. Hum. Genet..

[20] Swanson-Wagner R.A., Eichten S.R., Kumari S., Tiffin P., Stein J.C., Ware D., Springer N.M. (2010). Pervasive gene content variation and copy number variation in maize and its undomesticated progenitor. Genome Res..

[21] Marroni F., Pinosio S., Morgante M. (2014). Structural variation and genome complexity: Is dispensable really dispensable?. Curr. Opin. Plant Biol..

[22] Manolio T.A., Collins F.S., Cox N.J., Goldstein D.B., Hindorff L.A., Hunter D.J., McCarthy M.I., Ramos E.M., Cardon L.R., Chakravarti A. (2009). Finding the missing heritability of complex diseases. Nature.

[23] Sherman R.M., Salzberg S.L. (2020). Pan-genomics in the human genome era. Nat. Rev. Genet..

[24] Korona R. (2011). Gene dispensability. Curr. Opin. Biotechnol..

[25] Yao W., Li G., Zhao H., Wang G., Lian X., Xie W. (2015). Exploring the rice dispensable genome using a metagenome-like assembly strategy. Genome Biol..

[26] Gerdol M., Moreira R., Cruz F., Gómez-Garrido J., Vlasova A., Rosani U., Venier P., Naranjo-Ortiz M.A., Murgarella M., Greco S. (2020). Massive gene presence-absence variation shapes an open pan-genome in the Mediterranean mussel. Genome Biol..

[27] Wang K., Hu H., Tian Y., Li J., Scheben A., Zhang C., Li Y., Wu J., Yang L., Fan X. (2021). The Chicken Pan-Genome Reveals Gene Content Variation and a Promoter Region Deletion in IGF2BP1 Affecting Body Size. Mol. Biol. Evol..

[28] Li Z., Liu X., Wang C., Li Z., Jiang B., Zhang R., Tong L., Qu Y., He S., Chen H. (2023). The pig pangenome provides insights into the roles of coding structural variations in genetic diversity and adaptation. Genome Res..

[29] Gabur I., Chawla H.S., Lopisso D.T., von Tiedemann A., Snowdon R.J., Obermeier C. (2020). Gene presence-absence variation associates with quantitative Verticillium longisporum disease resistance in Brassica napus. Sci. Rep..

[30] Liu Y., Du H., Li P., Shen Y., Peng H., Liu S., Zhou G.A., Zhang H., Liu Z., Shi M. (2020). Pan-Genome of Wild and Cultivated Soybeans. Cell.

[31] Sun X., Jiao C., Schwaninger H., Chao C.T., Ma Y., Duan N., Khan A., Ban S., Xu K., Cheng L. (2020). Phased diploid genome assemblies and pan-genomes provide insights into the genetic history of apple domestication. Nat. Genet..

[32] Song J.M., Guan Z., Hu J., Guo C., Yang Z., Wang S., Liu D., Wang B., Lu S., Zhou R. (2020). Eight high-quality genomes reveal pan-genome architecture and ecotype differentiation of Brassica napus. Nat. Plants.

[33] Gao L., Gonda I., Sun H., Ma Q., Bao K., Tieman D.M., Burzynski-Chang E.A., Fish T.L., Stromberg K.A., Sacks G.L. (2019). The tomato pan-genome uncovers new genes and a rare allele regulating fruit flavor. Nat. Genet..

[34] Saco A., Rey-Campos M., Gallardo-Escárate C., Gerdol M., Novoa B., Figueras A. (2023). Gene presence/absence variation in Mytilus galloprovincialis and its implications in gene expression and adaptation. iScience.

[35] Bickhart D.M., Hutchison J.L., Null D.J., VanRaden P.M., Cole J.B. (2016). Reducing animal sequencing redundancy by preferentially selecting animals with low-frequency haplotypes. J. Dairy Sci..

[36] Bolger A.M., Lohse M., Usadel B. (2014). Trimmomatic: A flexible trimmer for Illumina sequence data. Bioinformatics.

[37] Rosen B.D., Bickhart D.M., Schnabel R.D., Koren S., Elsik C.G., Tseng E., Rowan T.N., Low W.Y., Zimin A., Couldrey C. (2020). De novo assembly of the cattle reference genome with single-molecule sequencing. Gigascience.

[38] Li H., Durbin R. (2009). Fast and accurate short read alignment with Burrows-Wheeler transform. Bioinformatics.

[39] (2019). “Picard Toolkit” Broad Institute, GitHub Repository. https://broadinstitute.github.io/picard/.

[40] Li H., Handsaker B., Wysoker A., Fennell T., Ruan J., Homer N., Marth G., Abecasis G., Durbin R., 1000 Genome Project Data Processing Subgroup (2009). The Sequence alignment/map (SAM) format and SAMtools. Bioinformatics.

[41] Pedersen B.S., Quinlan A.R. (2018). Mosdepth: Quick coverage calculation for genomes and exomes. Bioinformatics.

[42] Garrick D.J., Taylor J.F., Fernando R.L. (2009). Deregressing estimated breeding values and weighting information for genomic regression analyses. Genet. Sel. Evol..

[43] Tay Fernandez C.G., Marsh J.I., Nestor B.J., Gill M., Golicz A.A., Bayer P.E., Edwards D. (2022). An SGSGeneloss-Based Method for Constructing a Gene Presence-Absence Table Using Mosdepth. Methods Mol. Biol..

[44] Golicz A.A., Martinez P.A., Zander M., Patel D.A., Van De Wouw A.P., Visendi P., Fitzgerald T.L., Edwards D., Batley J. (2015). Gene loss in the fungal canola pathogen Leptosphaeria maculans. Funct. Integr. Genom..

[45] Mi H., Muruganujan A., Huang X., Ebert D., Mills C., Guo X., Thomas P.D. (2019). Protocol Update for large-scale genome and gene function analysis with the PANTHER classification system (v.14.0). Nat. Protoc..

[46] Bradbury P.J., Zhang Z., Kroon D.E., Casstevens T.M., Ramdoss Y., Buckler E.S. (2007). TASSEL: Software for association mapping of complex traits in diverse samples. Bioinformatics.

[47] Wang T., Birsoy K., Hughes N.W., Krupczak K.M., Post Y., Wei J.J., Lander E.S., Sabatini D.M. (2015). Identification and characterization of essential genes in the human genome. Science.

[48] Blomen V.A., Májek P., Jae L.T., Bigenzahn J.W., Nieuwenhuis J., Staring J., Sacco R., van Diemen F.R., Olk N., Stukalov A. (2015). Gene essentiality and synthetic lethality in haploid human cells. Science.

[49] Pons C., van Leeuwen J. (2023). Meta-analysis of dispensable essential genes and their interactions with bypass suppressors. Life Sci. Alliance.

[50] Crysnanto D., Leonard A.S., Fang Z.H., Pausch H. (2021). Novel functional sequences uncovered through a bovine multiassembly graph. Proc. Natl. Acad. Sci. USA.

[51] Lanier L.L. (2009). DAP10- and DAP12-associated receptors in innate immunity. Immunol. Rev..

[52] Khan M.Z., Khan A., Xiao J., Ma Y., Ma J., Gao J., Cao Z. (2020). Role of the JAK-STAT Pathway in Bovine Mastitis and Milk Production. Animals.

[53] Zozulya S., Echeverri F., Nguyen T. (2001). The human olfactory receptor repertoire. Genome Biol..

[54] Glusman G., Yanai I., Rubin I., Lancet D. (2001). The complete human olfactory subgenome. Genome Res..

[55] Olender T., Lancet D., Nebert D.W. (2008). Update on the olfactory receptor (OR) gene superfamily. Hum. Genom..

[56] Hasin-Brumshtein Y., Lancet D., Olender T. (2009). Human olfaction: From genomic variation to phenotypic diversity. Trends Genet..

[57] MacArthur D.G., Balasubramanian S., Frankish A., Huang N., Morris J., Walter K., Jostins L., Habegger L., Pickrell J.K., Montgomery S.B. (2012). A systematic survey of loss-of-function variants in human protein-coding genes. Science.

[58] Lee K., Nguyen D.T., Choi M., Cha S.Y., Kim J.H., Dadi H., Seo H.G., Seo K., Chun T., Park C. (2013). Analysis of cattle olfactory subgenome: The first detail study on the characteristics of the complete olfactory receptor repertoire of a ruminant. BMC Genom..

[59] Trimmer C., Keller A., Murphy N.R., Snyder L.L., Willer J.R., Nagai M.H., Katsanis N., Vosshall L.B., Matsunami H., Mainland J.D. (2019). Genetic variation across the human olfactory receptor repertoire alters odor perception. Proc. Natl. Acad. Sci. USA.

[60] Keller A., Zhuang H., Chi Q., Vosshall L.B., Matsunami H. (2007). Genetic variation in a human odorant receptor alters odour perception. Nature.

[61] McRae J.F., Mainland J.D., Jaeger S.R., Adipietro K.A., Matsunami H., Newcomb R.D. (2012). Genetic variation in the odorant receptor OR2J3 is associated with the ability to detect the “grassy” smelling odor, cis-3-hexen-1-ol. Chem. Senses.

[62] Arthur P.F., Archer J.A., Johnston D.J., Herd R.M., Richardson E.C., Parnell P.F. (2001). Genetic and phenotypic variance and covariance components for feed intake, feed efficiency, and other postweaning traits in Angus cattle. J. Anim. Sci..

[63] Zhou Y., Connor E.E., Wiggans G.R., Lu Y., Tempelman R.J., Schroeder S.G., Chen H., Liu G.E. (2018). Genome-wide copy number variant analysis reveals variants associated with 10 diverse production traits in Holstein cattle. BMC Genom..

[64] Ma Y., Liu S., Gao J., Chen C., Zhang X., Yuan H., Chen Z., Yin X., Sun C., Mao Y. (2021). Genome-wide analysis of pseudogenes reveals HBBP1’s human-specific essentiality in erythropoiesis and implication in β-thalassemia. Dev. Cell.

[65] Pink R.C., Wicks K., Caley D.P., Punch E.K., Jacobs L., Carter D.R. (2011). Pseudogenes: Pseudo-functional or key regulators in health and disease?. RNA.

[66] Choi C., Bae J., Kim S., Lee S., Kang H., Kim J., Bang I., Kim K., Huh W.K., Seok C. (2023). Understanding the molecular mechanisms of odorant binding and activation of the human OR52 family. Nat. Commun..

[67] Geithe C., Andersen G., Malki A., Krautwurst D. (2015). A Butter Aroma Recombinate Activates Human Class-I Odorant Receptors. J. Agric. Food Chem..

[68] Fleischer J., Bumbalo R., Bautze V., Strotmann J., Breer H. (2015). Expression of odorant receptor Olfr78 in enteroendocrine cells of the colon. Cell Tissue Res..

[69] Julliard A.K., Al Koborssy D., Fadool D.A., Palouzier-Paulignan B. (2017). Nutrient Sensing: Another Chemosensitivity of the Olfactory System. Front. Physiol..

[70] Connor E.E., Zhou Y., Liu G.E. (2018). The essence of appetite: Does olfactory receptor variation play a role?. J. Anim. Sci..

[71] Veerkamp R.F., Coffey M., Berry D., de Haas Y., Strandberg E., Bovenhuis H., Calus M., Wall E. (2012). Genome-wide associations for feed utilisation complex in primiparous Holstein-Friesian dairy cows from experimental research herds in four European countries. Animal.

[72] Ramirez-Diaz J., Cenadelli S., Bornaghi V., Bongioni G., Montedoro S.M., Achilli A., Capelli C., Rincon J.C., Milanesi M., Passamonti M.M. (2023). Identification of genomic regions associated with total and progressive sperm motility in Italian Holstein bulls. J. Dairy. Sci..

[73] Purfield D.C., Evans R.D., Berry D.P. (2019). Reaffirmation of known major genes and the identification of novel candidate genes associated with carcass-related metrics based on whole genome sequence within a large multi-breed cattle population. BMC Genom..

[74] Jalil Sarghale A., Moradi Shahrebabak M., Moradi Shahrebabak H., Nejati Javaremi A., Saatchi M., Khansefid M., Miar Y. (2020). Genome-wide association studies for methane emission and ruminal volatile fatty acids using Holstein cattle sequence data. BMC Genet..

[75] Gurao A., Kashyap S.K., Singh R. (2017). β-defensins: An innate defense for bovine mastitis. Vet. World.

[76] Izadpanah A., Gallo R.L. (2005). Antimicrobial peptides. J. Am. Acad. Dermatol..

[77] Costa A., Schwarzenbacher H., Mészáros G., Fuerst-Waltl B., Fuerst C., Sölkner J., Penasa M. (2019). On the genomic regions associated with milk lactose in Fleckvieh cattle. J. Dairy Sci..

[78] Atashi H., Chen Y., Wilmot H., Bastin C., Vanderick S., Hubin X., Gengler N. (2023). Single-step genome-wide association analyses for selected infrared-predicted cheese-making traits in Walloon Holstein cows. J. Dairy Sci..

[79] Vassilatis D.K., Hohmann J.G., Zeng H., Li F., Ranchalis J.E., Mortrud M.T., Brown A., Rodriguez S.S., Weller J.R., Wright A.C. (2003). The G protein-coupled receptor repertoires of human and mouse. Proc. Natl. Acad. Sci. USA.

[80] Lala T., Hall R.A. (2022). Adhesion G protein-coupled receptors: Structure, signaling, physiology, and pathophysiology. Physiol. Rev..

[81] Matmati M., Pouwels W., van Bruggen R., Jansen M., Hoek R.M., Verhoeven A.J., Hamann J. (2007). The human EGF-TM7 receptor EMR3 is a marker for mature granulocytes. J. Leukoc. Biol..

[82] Benjamin A.L., Green B.B., Crooker B.A., McKay S.D., Kerr D.E. (2016). Differential responsiveness of Holstein and Angus dermal fibroblasts to LPS challenge occurs without major differences in the methylome. BMC Genom..

[83] Buaban S., Lengnudum K., Boonkum W., Phakdeedindan P. (2022). Genome-wide association study on milk production and somatic cell score for Thai dairy cattle using weighted single-step approach with random regression test-day model. J. Dairy. Sci..

