# Supplementary material for: A Pilot Detection and Associate Study of Gene Presence-Absence Variation in Holstein Cattle
Source: Animals (Basel). 2024 Jun 28;14(13):1921. doi: 10.3390/ani14131921 (PMC11240624; doi:10.3390/ani14131921)
Supplement: Supplementary file 1 [file animals-14-01921-s001.zip › animals-3043907 supplement fig.docx]

**Supplementary Figures**

**Supplementary Figure 1.** (A) Principal component analysis based on gene PAV matrix of 173 Holstein animals. (B) Neighbor-joining phylogenetic tree constructed based on gene PAV matrix of 173 Holstein animals.
